# Transcriptomic changes in donor soybean, dodder bridge, and the connected recipient soybean induced by cadmium addition

**DOI:** 10.3389/fpls.2025.1567412

**Published:** 2025-04-17

**Authors:** Hangkai Pan, Li Zhou, Junmin Li

**Affiliations:** ^1^ Zhejiang Key Laboratory for Restoration of Damaged Coastal Ecosystems, School of Life Sciences, Taizhou University, Taizhou, China; ^2^ Zhejiang Provincial Key Laboratory of Plant Evolutionary Ecology and Conservation, School of Life Sciences, Taizhou University, Taizhou, China; ^3^ Engineering Research Center of Agricultural Microbiology Technology, Ministry of Education & Heilongjiang Provincial Key Laboratory of Plant Genetic Engineering and Biological Fermentation Engineering for Cold Region & Key Laboratory of Molecular Biology, College of Heilongjiang Province & School of Life Sciences, Heilongjiang University, Harbin, China; ^4^ School of Advanced Science, Taizhou University, Taizhou, China

**Keywords:** cadmium, dodder bridge, transcriptomic analysis, mobile mRNAs, indirect effect

## Abstract

**Background:**

*Cuscuta* spp. (dodders) are parasitic plants that belong to the Convolvulaceae family. In nature, dodder often forms a bridge-like connection between two or more host plants like, which is known as a dodder bridge. Cadmium (Cd^2+^) is an important heavy metal ion that affects plant growth. However, it remains unclear whether Cd^2+^ treatment can directly or indirectly induce transcriptomic changes in plants through dodder bridge.

**Results:**

In this study, a pot experiment was conducted to investigate the effects of Cd^2+^ treatment on donor plant and neighboring recipient plant connected by dodder bridge. Transcriptome analysis revealed that Cd^2+^ treatment significantly affected the expression of genes involved in the ‘Plant-pathogen interaction’, ‘phenylpropanoid biosynthesis’, and ‘isoflavonoid biosynthesis’ pathways in both donor and recipient plants at 2, 12, 24, and 48 h. Cd^2+^ indirectly induced changes in the dodder bridge, which included processes related to oxidation-reduction (‘oxidation-reduction process’, ‘oxidoreductase activity’, and ‘regulation of transcription’) and Ca^2+^ signaling pathways (‘Plant-pathogen interaction’, ‘MAPK signaling pathway’, ‘AMPK signaling pathway’, ‘mTOR signaling pathway’). Additionally, mRNA transfer was observed from soybean to dodder. mRNA, Ca^2+^ and ROS might play crucial roles in the signal transduction process induced by Cd^2+^ stress.

**Conclusion:**

Cd^2+^ treatment could directly and indirectly induce transcriptomic changes in the donor plant and neighboring recipient plant connected by dodder bridge. These results contribute to a better understanding of how plants connected by dodder bridges respond to environmental stresses.

## 1 Background

Heavy metal pollution in soil has gradually increased by industrial and agricultural development (Thornton and Webb, 1980). Cadmium (Cd^2+^) is one of the key heavy metals to contaminate soil (Qiu et al., 2011). Cd^2+^ may not only be taken up by plants, but may also be subsequently transferred to the food chain (Li et al., 2023; Sterckeman and Thomine, 2020). For example, Cd^2+^ could be biologically amplified in herbivores than grass (Hunter and Johnson, 1982). Compared to the soil-plant-herbivore system, soil-plant-parasitic plant system has received less attention in the research of tropic transfer of Cd^2+^ (Zagorchev et al., 2021).

*Cuscuta* spp. (Convolvulaceae) are holoparasitic plants that obtain water and nutrients through haustorium (Revill et al., 2005). It has been well documented that various molecules, including proteins (Haupt et al., 2001; Jiang et al., 2013), amino acids, ions (Hibberd and Jeschke, 2001), viroids (Dorst and Peters, 1974; Birschwilks et al., 2006), and nucleic acids (Mower et al., 2010; Westwood and Kim, 2017; Wu, 2018) can be exchanged from host to *Cuscuta* spp. through haustorial junctions. Although studies of Cd^2+^ stress on the growth of *Cuscuta* spp. are limited (Zagorchev et al., 2021), Chen et al. (2021) found that Cd^2+^ could be transferred from host to *Cuscuta chinensis*. However, the transfer of Cd^2+^ from soybean plants to *C. chinensis* is limited with the Cd^2+^ transfer coefficient being significantly lower than one. Zhang et al. (2024) reported that nitrate and phosphate deficiencies could affect the interplant transport of mRNAs and proteins between soybean and dodder plants. In nature, *Cuscuta* spp. often referred to as dodders, frequently connect two or more host plants to form dodder bridge. Through these bridges, molecules, such as sucrose, Potato Virus Y (PVY), and phytoplasma, could be transferred from host plant to the neighboring plants (Marcone et al., 1997; Hibberd and Jeschke, 2001; Birschwilks et al., 2006). However, little is known about the transport of Cd^2+^ from host donor plant to neighboring recipient plant through dodder bridge.

Recent studies have demonstrated that stress on donor plants can induce transcriptomic changes in neighboring recipient plants connected by dodder bridge (Hettenhausen et al., 2017; Li et al., 2020; Zhang et al., 2021). For example, Hettenhausen et al. (2017) found that herbivory treatment could induce phytohormone pathways in neighboring maize plants, such as ‘response to jasmonic acid’, ‘ethylene metabolic’, and ‘salicylic acid metabolic’, as well as secondary metabolic processes including ‘phenylpropanoid metabolic’, ‘lignin metabolic’, ‘olefin metabolic’, and ‘monoterpene metabolic’ changes. Li et al. (2020) observed that salt treatment could induce the neighboring cucumber with ‘single-organism carbohydrate metabolic process’, ‘carbohydrate biosynthetic process’, and ‘oxidoreduction coenzyme metabolic process’ changes. It can be hypothesized that Cd^2+^ stress may also induce transcriptomic changes in neighboring plants connected by dodder bridge. Additionally, previous research has shown that dodders express the antioxidant enzyme-related genes indirectly induced by the Cd^2+^ exposure on the host plant (Vurro et al., 2011). However, there is a significant gap regarding experimental comparison between the responses of donor plant, dodder and neighboring recipient plant to Cd^2+^ stress.

In this study, *Cuscuta gronovii* was used to parasitize two soybean plants simultaneously to form dodder bridge. Transcriptomic analysis was performed on donor soybean, dodder bridge and neighboring recipient soybean after the donor soybean was treated with Cd^2+^. The aim of this study was to investigate following questions: 1) Whether the Cd^2+^ stress on the donor soybean could indirectly induce transcriptomic changes in the dodder bridge? Are there any differences with the duration of treatment? 2) Whether the Cd^2+^ stress on the donor soybean could indirectly induce transcriptomic changes in the neighboring recipient soybean? Are there any differences with the duration of treatment? 3) Whether the Cd^2+^ stress on the donor soybean could affect the mobile mRNA between the donor plants and the dodder bridge or between the dodder bridge and the recipient plants?

## 2 Results

### 2.1 Changes in donor soybean directly induced by Cd^2+^ addition

**Figure 1:**
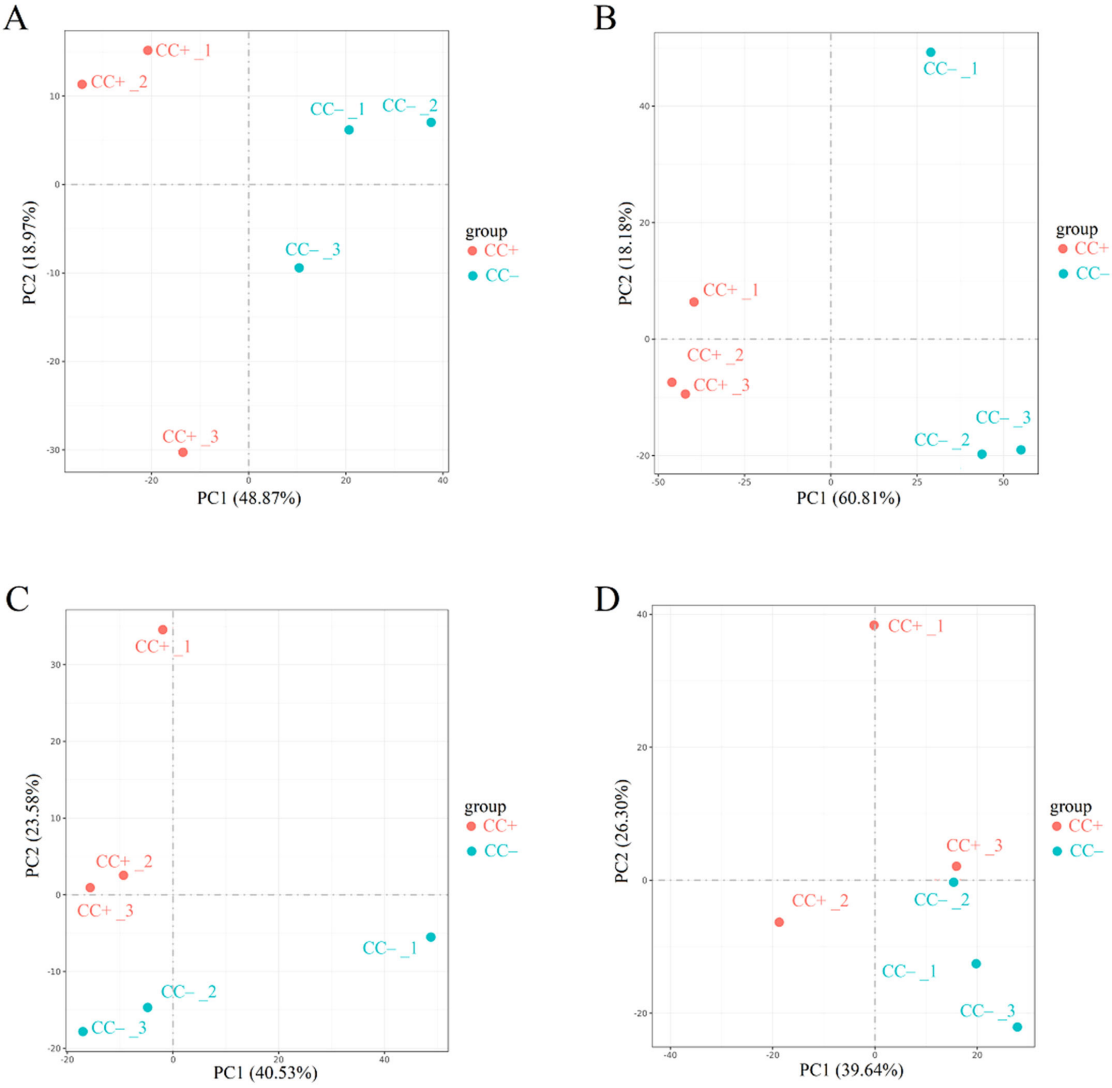
Results of PCA analysis between CC^+^ and CC^–^ at 2 h **(A)**, 12 h **(B)**, 24 h **(C)**, and 48 h **(D)**. Different numbers indicate different replicates. PC1 and PC2 indicate the first and second principal components. The percentage in brackets indicates the percentage of variance explained by PC1 or PC2.

**Figure 2:**
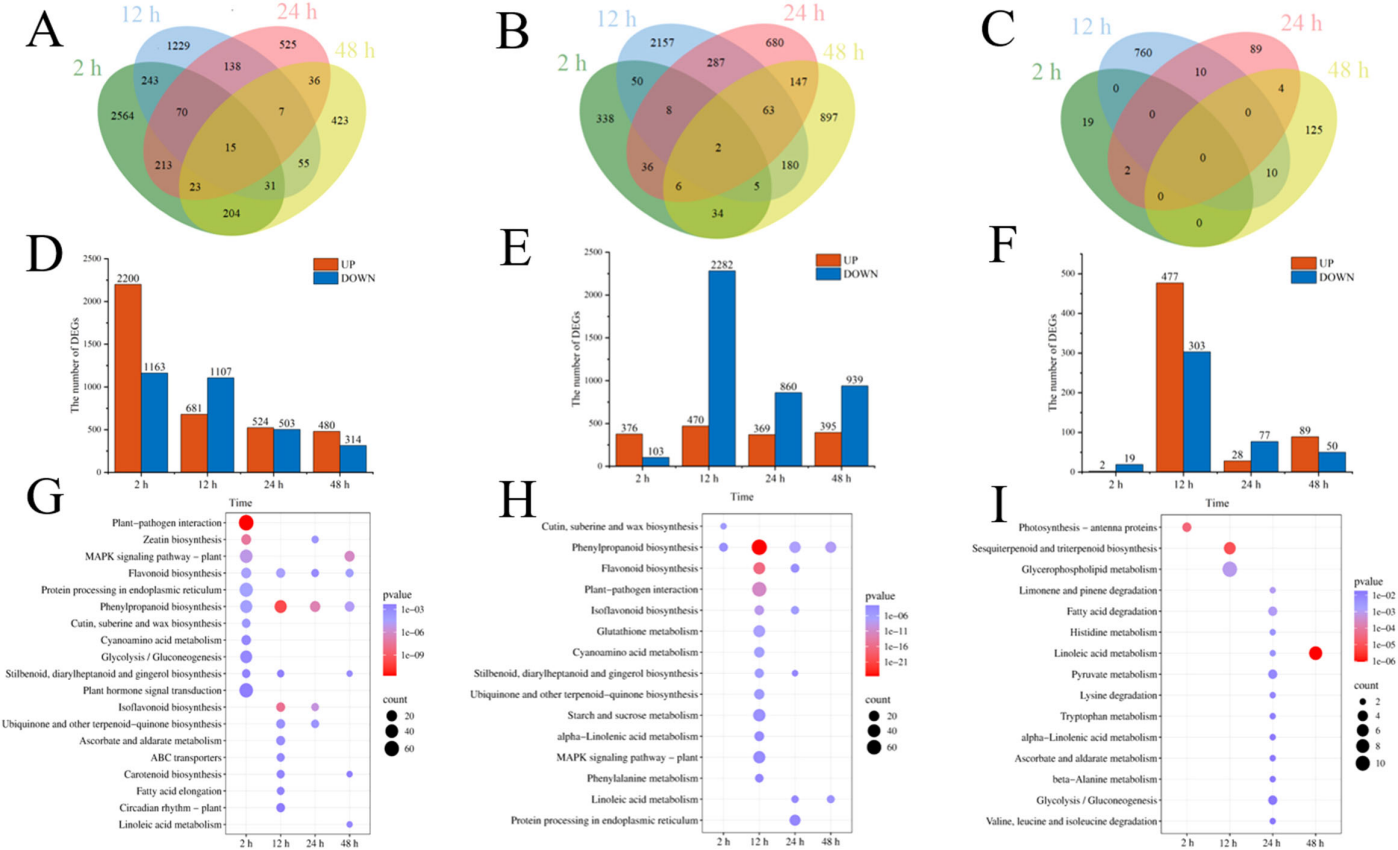
Venn diagram of DEGs **(A–C)**, the number of up and down regulated DEGs **(D–F)**, and KEGG pathway enrichment of DEGs **(G–I)** between CC^+^ and CC^–^ **(A, D, G)**, TC– and CC– **(B, E, H)**, and TC^+^ and TC^–^ **(C, F, I)** at 2 h, 12 h, 24 h, and 48 h. Color of the point represents size of p-value. The number of differential genes included in each pathway is expressed by the point’s size.

**Figure 3:**
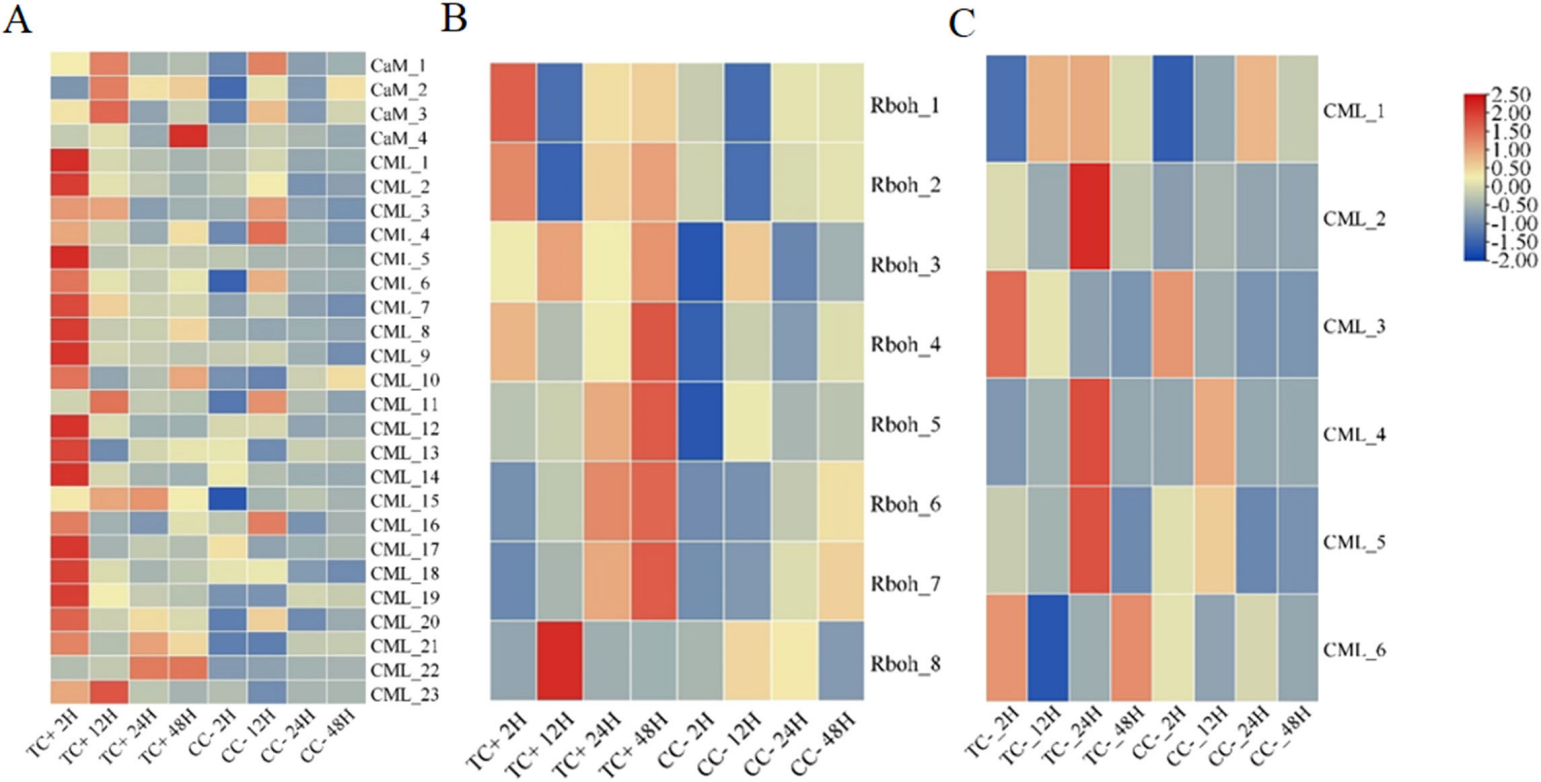
The heatmaps of the express of CaM and CML **(A)** and Rboh **(B)** genes between TC^+^ and CC^–^ soybeans, and CML genes **(C)** between TC^–^ and CC^–^ soybeans after Cd^2+^ treatment at 2 h, 12 h, 24 h, and 48 h.

Cd^2+^ was not detected in leaves, stems, and roots of TC^–^ and CC^–^ soybean (**Supplementary Figure S1**). Principal Component Analysis (PCA) of transcripts between CC^+^ and CC^–^ treatments showed that Cd^2+^ addition directly induced significant changes at the transcriptomic level in donor soybean at 2, 12, 24, and 48 h (**Figure 1**). Venn diagram showed that the variation of the number of DEGs between CC^+^ and CC^–^at 2 h, 12 h, 24 h and 48 h were high (**Figure 2A**). At 2, 12, 24, and 48 h, DEGs between CC^+^ and CC^–^ leaves were 3363 (2200 up-regulated and 1163 down-regulated), 1788 (681 up-regulated and 1107 down-regulated), 1024 (524 up-regulated and 503 down-regulated), and 794 (480 up-regulated and 314 down-regulated), respectively (**Figure 2D**). The top 19 enriched pathways of DEGs between CC^+^ and CC^–^ at 2, 12, 24, and 48 h were shown in **Figure 2G**. Notably, ‘flavonoid biosynthesis’ and ‘phenylpropanoid biosynthesis’ pathways were enriched at four time points (**Figure 2G**). The KEGG pathways enriched among the up-regulated DEGs between CC^+^ and CC^-^ include ‘phenylpropanoid biosynthesis,’ ‘flavonoid biosynthesis’ ‘isoflavonoid biosynthesis’ and ‘plant-pathogen interaction’ which are consistently significantly enriched after Cd^2+^ treatment (**Supplementary Figure S2**; **Supplementary Table S1**). The total of 15 DEGs were shared between CC^+^ and CC^–^ leaves at 2, 12, 24, and 48 h, including *WRKY transcription factor 12*, *WRKY transcription factor 50*, *pathogenesis-related protein (PR10)*, *expansin 4 (EXP4)*, *UDP-glycosyltransferase 88A1*, *putative receptor protein kinase ZmPK1*, and *glutamate receptor 2.9* (**Supplementary Table S2**). Considering genes related to Ca^2+^ and ROS mediated signaling transduction, 4 Calmodulin (CaM) genes and 23 Calmodulin-like proteins (CML) genes were found to be up-regulated in TC^+^ compared to CC^–^ (**Figure 3A**). Additionally, 8 genes related to respiratory burst oxidase homologue (rboh) were upregulated in TC^+^, but not in TC^–^, compared to CC^–^ (**Figure 3B**).

### 2.2 Changes in neighboring recipient soybean indirectly induced by Cd^2+^addition

**Figure 4:**
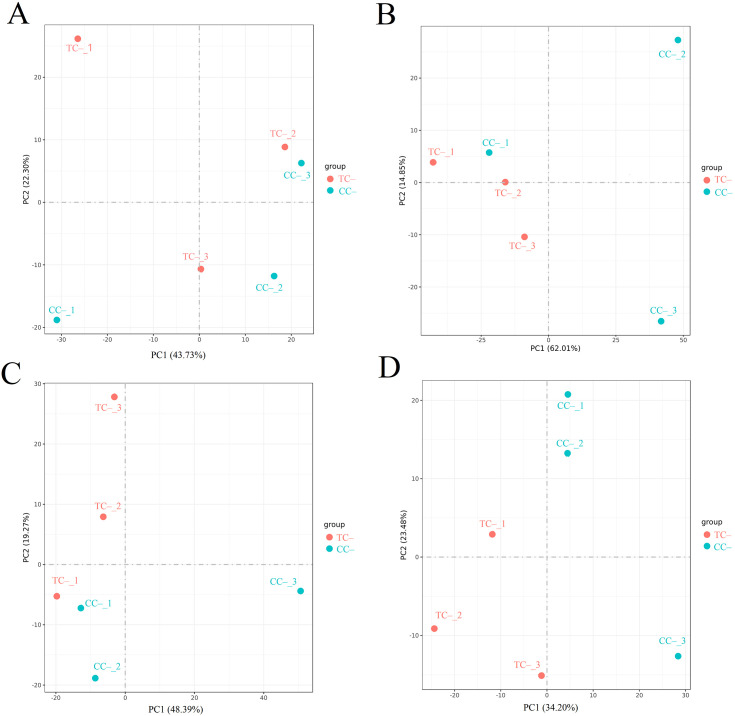
Results of PCA analysis between TC^–^ and CC^–^ at 2 h **(A)**, 12 h **(B)**, 24 h **(C)**, and 48 h **(D)**. Different numbers indicate different replicates. PC1 and PC2 indicate the first and second principal components. The percentage in brackets indicates the percentage of variance explained by PC1 or PC2.

Comparison of the transcriptomic data between TC^–^ and CC^–^ can reveal the indirect response of the recipient soybean to the addition of Cd^2+^. PCA of transcripts between TC^–^ and CC^–^ treatments showed that Cd^2+^ addition indirectly induced significant changes at the transcriptomic level in recipient soybean at 2, 12, 24, and 48 h (**Figure 4**). Venn diagram showed that the variation of the number of DEGs between TC^–^ and CC^–^at 2 h, 12 h, 24 h and 48 h were high (**Figure 2B**). At 2, 12, 24 to 48 h, DEGs between TC^–^ and CC^–^ leaves were 479 (376 up-regulated and 103 down-regulated), 2752 (470 up-regulated and 2282 down-regulated), 1229 (369 up-regulated and 860 down-regulated), and 1334 (395 up-regulated and 939 down-regulated), respectively (**Figure 2E**). The top 15 enriched pathways of DEGs between TC^–^ and CC^–^ at 2, 12, 24, and 48 h were shown in **Figure 2H**. ‘Phenylpropanoid biosynthesis’ pathway was enriched between TC^–^ and CC^–^ at 2, 12, 24, to 48 h (**Figure 2H**; **Supplementary Table S3**).

**Figure 5:**
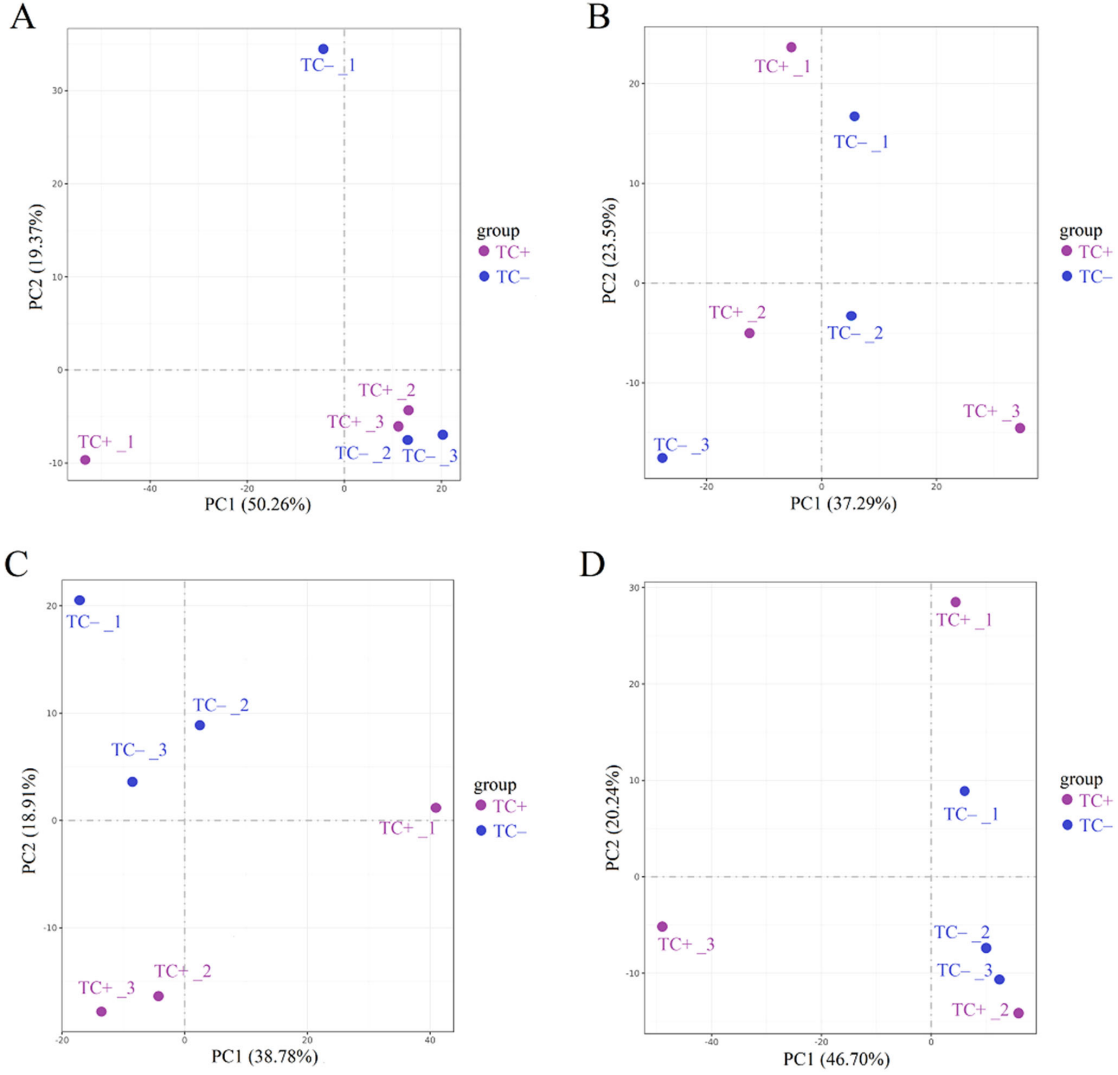
Results of PCA analysis between TC^+^ and TC^–^ at 2 h **(A)**, 12 h **(B)**, 24 h **(C)**, and 48 h **(D)**. Different numbers indicate different replicates. PC1 and PC2 indicate the first and second principal components. The percentage in brackets indicates the percentage of variance explained by PC1 or PC2.

Comparison of the transcriptomic data between TC^+^ and TC^–^ can reveal the difference between direct and indirect responses of donor and recipient soybean to Cd^2+^ addition. PCA results of transcripts between TC^+^ and TC^–^ treatments showed that the direct responses of donor soybean to Cd^2+^ addition were similar to the indirect responses of the recipient soybean at 2, 12, 24, and 48 h (**Figure 5**). Venn diagram showed that the number of DEGs between TC^–^ and TC^–^at 2 h, 12 h, 24 h and 48 h were lower than those between CC^+^ and CC^–^, and TC^–^ and CC^–^ (**Figures 2A-C**). The number of DEGs between TC^+^ and TC^–^ leaves was 21 (2 up-regulated and 19 down-regulated), 780 (477 up-regulated and 303 down-regulated), 105 (28 up-regulated and 77 down-regulated), and 139 (89 up-regulated and 50 down-regulated) after Cd^2+^ treatment for 2, 12, 24, and 48 h, respectively (**Figure 2F**). The top 15 enriched pathways of DEGs between CC^+^ and CC^–^ at 2, 12, 24, and 48 h were shown in **Figure 2I**. ‘Photosynthesis’ and ‘Photosynthesis - antenna proteins’ pathways were enriched significantly between TC^+^ and TC^–^ at 2 h (**Figure 2I**), indicating the sensitivity of photosynthesis pathway to Cd^2+^ addition. ‘Sesquiterpenoid and triterpenoid biosynthesis’ and ‘glycerophospholipid metabolism’ pathways were enriched significantly between TC^+^ and TC^–^ at 12 h (**Figure 2I**) and ‘Linoleic acid metabolism’ pathway was enriched significantly between TC^+^ and TC^–^ at 48 h (**Figure 2I**), indicating the responses of chemical responses to Cd^2+^ addition. Considering genes related to Ca^2+-^mediated signaling transduction, 6 CML genes exhibited increased expression in TC^-^ compared to CC^-^ (**Figure 3C**), while no DEGs related with CaM were found.

### 2.3 Changes in dodder bridge indirectly induced by Cd^2+^ addition

**Figure 6:**
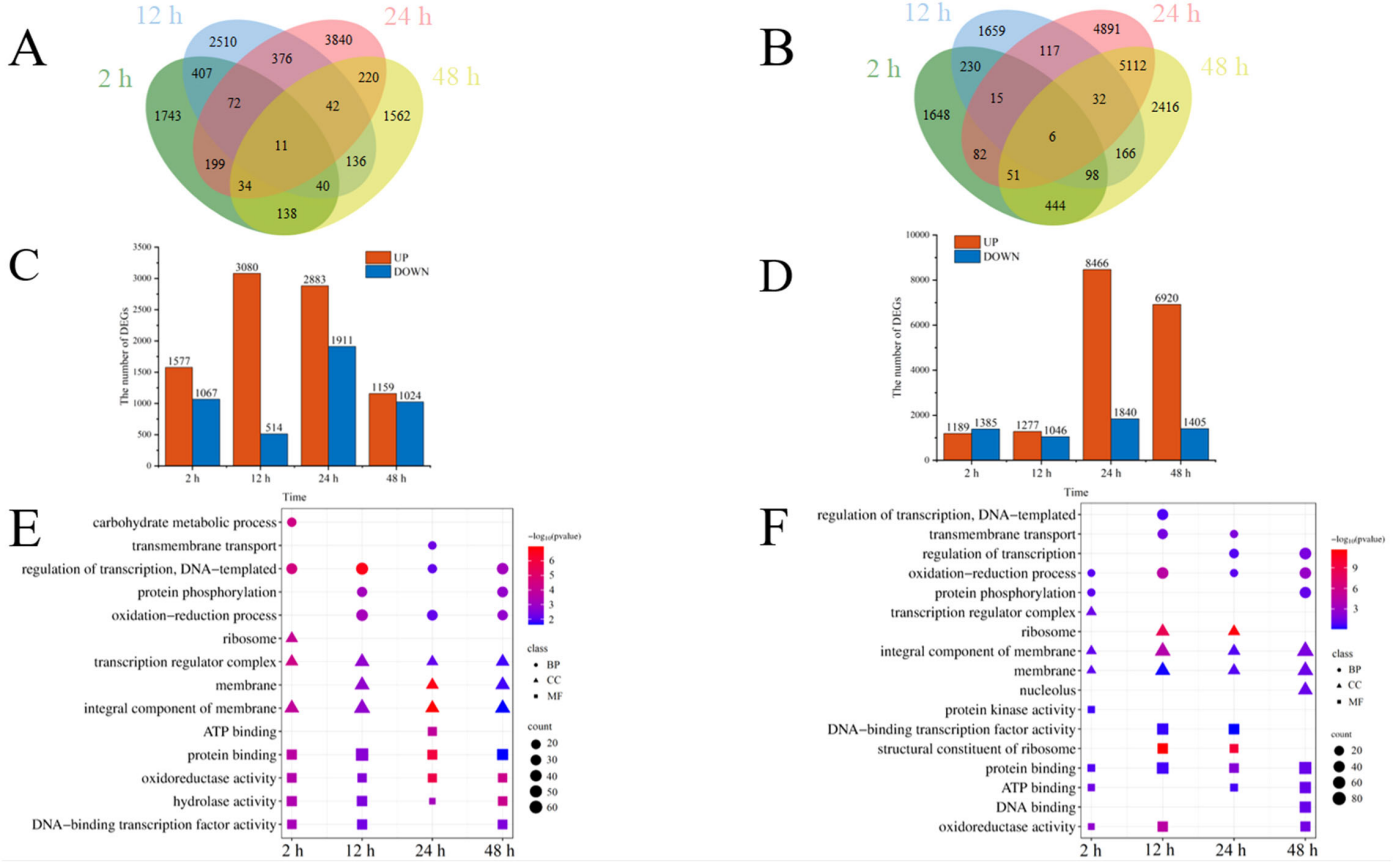
Venn diagram of DEGs **(A, B)**, the number of up and down regulated DEGs **(C, D)**, and GO enrichment of DEGs **(E, F)** of dodder bridge between PControl dodders and NControl dodders **(A, C, E)**, and between Treated dodders and NControl dodders **(B, D, F)** at 2, 12, 24, and 48 h. Color of the point represents the size of *p*-value. The number of differential genes included in each process is expressed by the point’s size.

Comparison of the transcriptomic data between PControl dodders and NControl dodders and between treated dodders and NControl dodders can reveal the response of dodder bridge to Cd^2+^ addition. Venn diagram showed the number of DEGs between PControl dodders and NControl dodders was similar with that between Treated dodders and NControl dodders at 2 and 12 h, but lower at 24 and 48 h (**Figures 6A, B**). Compared to the NControl dodders, Cd^2+^ addition in PControl dodders resulted in 2644 (1577 up-regulated and 1067 down-regulated), 3594 (3080 up-regulated and 514 down-regulated), 4744 (2883 up-regulated and 1911 down-regulated), and 2183 (1159 up-regulated and 1024 down-regulated) DEGs in dodder bridge at 2, 12, 24 and 48 h, respectively (**Figure 6C**); however, Cd^2+^ addition in Treated dodders induced 2574 (1189 up-regulated and 1385 down-regulated), 2323 (1277 up-regulated and 1046 down-regulated), 10306 (8466 up-regulated and 1840 down-regulated), and 8325 (6920 up-regulated and 1405 down-regulated) DEGs in dodder bridge at 2 h, 12 h, 24 h and 48 h, respectively (**Figure 6D**). GO enrichment results showed that, compared to NControl dodders, most of the DEGs were both enriched in ‘oxidation-reduction process’, ‘oxidoreductase activity’, and ‘regulation of transcription’ in positive control dodders (**Figure 6E**) and treated dodders (**Figure 6F**). KEGG enrichment results showed that the up-regulated DEGs (Treated dodders vs NControl dodders) were enriched in ‘plant-pathogen interaction’ and ‘MAPK signaling pathway – plant’ pathway at 12, 24 and 48 h. ‘biosynthesis of secondary metabolites’, ‘metabolic pathways’ and ‘phenylpropanoid biosynthesis’ were enriched at 2 and 12 h. And ‘AMPK signaling pathway’, ‘oxidative phosphorylation’, and ‘mTOR signaling pathway’ were enriched at 24 and 48 h (**Supplementary Figure S4**; **Supplementary Table S4**).

### 2.4 Interplant mobile mRNAs from soybean to dodder bridge

**Figure 7:**
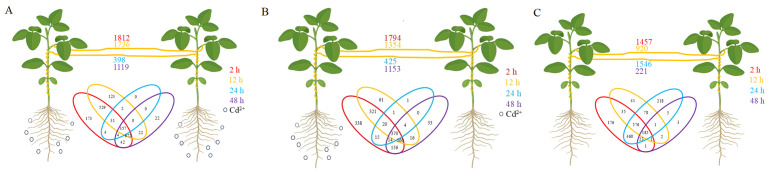
Numbers of mobile mRNAs in dodder bridge after Cd^2+^ treatment for 2 h (red), 12 h (ochre), 24 h (blue), and 48 h (purple). **(A)** Positive control. Both soybeans were treated with 100 mL 200 mg/L CdSO_4_. **(B)** Experimental treatment. One soybean was treated with 100 mL 200 mg/L CdSO_4_ and the other was treated with ddH_2_O. **(C)** Negative control. Both soybeans were treated with ddH_2_O. Venn diagram represents the mobile soybean mRNAs detected in dodders at 2 h (red), 12 h (ochre), 24 h (blue), and 48 h (purple). Black circles represent Cd^2+^ addition.

**Figure 8:**
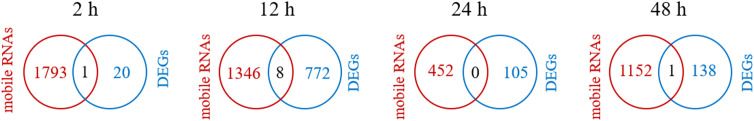
Venn diagram analysis of mobile mRNAs detected in dodder bridge (red circle) and DEGs between neighboring recipient soybean in TC^+^ and TC^–^ treatments (blue circle) at 2 h, 12 h, 24 h, and 48 h.

To investigate the effect of Cd^2+^ stress on transfer of mobile mRNA from soybean to dodder bridge, genes were identified as mobile mRNA on the reference genome annotated only to soybean. A total of 2569 mobile soybean mRNAs were identified (**Supplementary Table S5**). In positive control, 1812, 1736, 398, and 1119 mRNA of soybean were transferred into dodders at 2 h, 12 h, 24 h, and 48 h, respectively (**Figure 7A**). In experimental treatment, 1794, 1354, 425, and 1153 mRNA of soybean were transferred into dodder at 2 h, 12 h, 24 h, and 48 h, respectively (**Figure 7B**). In negative control, 1457, 920, 1546, and 221 mRNA of soybean were transferred into dodder at 2 h, 12 h, 24 h, and 48 h, respectively (**Figure 7C**). Additionally, some transcription factor proteins had been identified, such as WRKY transcription factors. A total of 259 genes were identified to be transferred from soybean to dodder after the addition of Cd^2+^ (**Supplementary Table S6**). Among the identified genes, one encodes a protein associated with heavy metal stress response, specifically the ‘metal transporter Nramp6 isoform X2’. Additionally, the transferred genes encompass a subset involved in plant stress response mechanisms, as well as others related to substance transport functions. The top five most abundant soybean genes detected in dodder bridge are as follows: ‘truncated transcription factor CAULIFLOWER A (GmCAULIFLOWER A)’, ‘unknown’, ‘cation/H(+) antiporter 20 (CHX20)’, ‘arogenate dehydratase/prephenate dehydratase 6, chloroplastic (ADT6)’, and ‘chalcone isomerase RNA (CHI RNA)’. Most of the DEGs between TC^+^ and TC^–^ were different from mobile mRNAs, for only 1, 8, 0, and 1 genes were found common at 2, 12, 24, and 48 h, respectively (**Figure 8**).

### 2.5 RT-qPCR verification

Six genes involved in Ca^2+^ and ROS signaling transferring systems were selected for RT-qPCR. The six genes exhibited high expression levels and were differentially expressed at TC^+^, TC^–^, and CC^–^ soybeans (**Supplementary Figure S5**). The validation results align with the expression patterns observed in the sequencing data, thereby confirming the reliability of the transcriptome data.

## 3 Discussion

It has been well documented that Cd^2+^ stress poses significant threat to the growth of plants (El-Esawi et al., 2020; Luo et al., 2023). For example, Cd^2+^ could affect antioxidant enzyme activity, malondialdehyde, chlorophyll, phenolic compounds, and flavonoid content in soybean leaves (El-Esawi et al., 2020). However, little is known about the indirect transcriptomic changes of neighboring recipient plants induced by dodder bridge. In this study, although Cd^2+^ was not transferred across the dodder bridge between two soybean plants, it was clear that the addition of Cd^2+^ can directly induce transcriptomic changes in the donor soybean and indirectly in the neighboring recipient soybean (**Figures 1**, **2**, **4**, **5**). In addition, we found that the indirectly induced changes reached peak at 12 h, which was later than those directly induced by Cd^2+^ addition (**Figure 2**). Transcriptomic analysis showed that high concentration of cadmium could have significant impact on soybean gene expression in 4 h and affect various stress-related pathways, including flavonoid synthesis, MAPK cascade, cell wall synthesis, and peroxidase activity (Gong et al., 2023; Xian et al., 2023). The delay suggested that it may need time for the systematic signals induced by Cd^2+^ addition across the dodder bridge from the donor soybean to the neighboring recipient soybean. In addition, the number of DEGs between TC^+^ and TC^–^ were less than those between CC^+^ and CC^–^, and TC^–^ and CC^–^ (**Figure 2**), suggesting the similarity of the direct and indirect responses of donor and recipient soybean to Cd^2+^ addition. So did the PCA analysis results (**Figures 1**, **4**, **5**).

In this study, KEGG pathways enrichment of DEGs between CC^+^ and CC^–^ (**Figure 2**) showed that most of the pathways were involved with signaling transduction at 2 h, while others were involved with the biosynthesis of secondary metabolites at 12 h, 24 h, and 48 h. These results indicated a shift in gene expression involved in early signal transduction to late defense responses. The KEGG pathway enrichment of up-regulated genes between CC^+^ and CC^–^ soybeans also showed that pathways such as ‘phenylpropanoid biosynthesis’ ‘flavonoid biosynthesis’ ‘isoflavonoid biosynthesis’ and ‘plant-pathogen interaction’ were both significantly enriched following Cd^2+^ treatment (**Supplementary Figure S2**; **Supplementary Table S1**), suggesting that Cd^2+^ is a stress to soybean and induced the chemical defense responses. These results were consistent with the previous reports (El-Esawi et al., 2020; Xian et al., 2023; Yang et al., 2024). Notably, ‘Plant-pathogen interaction’, ‘phenylpropanoid biosynthesis’, and ‘isoflavonoid biosynthesis’ pathways were both enriched between CC^+^ and CC^–^, TC^–^ and CC^–^ at four time points (**Supplementary Figure S3**; **Supplementary Table S3**), but was not enriched between TC^+^ and TC^–^. This indicated these pathways might be conserved in the soybean’s initiation response to Cd^2+^ induced systemic signals, and the effects of Cd^2+^ addition on these pathways in TC^+^ and TC^–^ were almost synchronous in time and had similar degrees of impact.

The molecules with signal transduction functions in plants include: plant hormones (auxins, abscisic acid, etc.), gaseous signal molecules (NO), Ca^2+^, ROS, and mRNA, etc (Demidchik et al., 2018). Firstly, the transferred mRNA might play roles in Cd^2+^ induced systemic signals. In plants, mRNA can move through the phloem vascular system to distant tissues to act as non-cell autonomous signals and exert effects outside the cells of origin (Yang et al., 2019). It has been well documented that mRNA can be transferred between host plant and parasitic *Cuscuta* spp (Kim et al., 2014; Song et al., 2022). The transcriptome of *Arabidopsis thaliana*, tomato (*Solanum lycopersicum*), and cucumber (*Cucumis sativus*) were found in dodders (Kim et al., 2014; Song et al., 2022). Recent evidence showed that nitrogen stress and herbivores feeding strongly affected the number and species of mobile mRNAs between host plants and dodders parasite (Zhang et al., 2021; Song et al., 2022). In this research, regardless of Cd^2+^ treatment or not, soybean’s mobile mRNA could be detected in dodder bridges, indicating the presence of mobile mRNA transferring between donor and recipient soybeans through dodder bridge. Additionally, we found that the expression of 13 Gm*WRKY* genes were up-regulated in dodders and 4 Gm*WRKY* were up-regulated in TC^–^ soybeans and TC^+^ soybeans (**Supplementary Table S5**, **Supplementary Figure S6**). Transcription factors WRKY also play essential roles in ‘phenylpropanoid biosynthesis’, ‘flavonoid synthesis’, and ‘isoflavonoid synthesis’ (Dong and Lin, 2021; Song et al., 2024). We also found the 4 Gm*WRKY* genes show a significant positive correlation with the differentially expressed genes in the phenylpropanoid biosynthesis pathway (TC^+^ vs CC^–^, TC^–^ vs CC^–^) (**Supplementary Figure S7**). Thus, *WRKY* mRNA could be a potential mRNA. Secondly, Ca^2+^ might play roles in Cd^2+^ induced systemic signals. Calcium-binding proteins such as CaM and CML play crucial roles in plant cells by modulating various cellular processes through their interaction with Ca^2+^ (Andrews et al., 2021). In our results, Cd^2+^ treatment not only induced the up-regulation of CaM and CML-related genes in CC^+^ soybeans, but also in the recipient TC^-^ soybean where the expression levels were similar to those in the donor soybean TC^+^. This suggested that Cd^2+^ treatment triggers the generation of Ca^2+^ cascade signals in TC^+^ and facilitated their transmission between the donor soybean and recipient soybean plants through enhancing the expression of CaM and CML proteins. Thirdly, ROS might play roles in Cd^2+^ induced systemic signals. ROS, acting as signaling molecules, participate in plant growth, development, and stress responses, interacting with Ca²^+^ signals to collectively enhance plant resistance (Choi et al., 2017). Ca²^+^ and ROS often act in concert in response to abiotic stress (Koster et al., 2022). There are some oxidases in plants, such as rboh, which can produce ROS that can act as signaling molecules to activate plant defense mechanisms (Bi et al., 2022). In this study, compared with CC^-^, 8 rboh genes were significantly up-regulated in TC^+^ soybeans, but not in TC^-^ soybeans. This indicated that after Cd²^+^ treatment, ROS produced in the donor plant TC^+^ soybeans might be transferred to TC^–^soybeans, while TC^–^soybeans without the directly treated by Cd^2+^ would do not produce ROS by themselves. However, all the above potential systemic signals and their functions might be recognized in the future studies.

Short exposure of low concentration of Cd^2+^ resulted in increased antioxidant activity in both dodders callus and seedlings (Srivastava et al., 2004). This indicated that, despite losing some genes associated with photosynthesis during evolution, it still required the participation of antioxidant enzyme in heavy metal detoxification, similar to higher plants (Srivastava et al., 2004). In this study, we also focused on the changes in the transcriptome of dodder after cadmium treatment. With Cd^2+^ treatment for 2 h, 12 h, 24 h, and 48 h, ‘regulation of transcription’ and ‘oxidation-reduction process’ pathways were both enriched. ‘Oxidoreductase activity’ pathway was enriched in the DEGs between treated dodders and negative control dodders at 2 h, 12 h, and 48 h. In addition, the Ca^2+^ plays a key role in the ‘AMPK signaling pathway’, ‘mTOR signaling pathway’, ‘MAPK signaling pathway – plant’ and ‘plant-pathogen interaction’ pathways, which were both enriched at 24 h and 48 h (**Supplementary Figure S4**) (Wurzinger et al., 2011; Mohanta et al., 2018; Tabassum and Blilou, 2022). This indicated that Ca^2+^ transferred from TC^+^ soybeans and affected the dodder genes expression after Cd^2+^ treatment. However, the Ca^2+^ influx of host plant into dodder and the signaling mechanisms responsible for the changes in oxidation-reduction related genes in dodder bridge remain unclear. Further studies should be performed focusing on the potential signaling and their consequent transduction.

## 4 Materials and methods

### 4.1 Plant material and culture

Soybean seeds (Zhonghuang13) were purchased from Jindou Seed Industry Co., Ltd, Liangshan County, China. On August 13, 2023, seeds were cultivated in a pot (20 cm × 25 cm × 25 cm) containing a mixed substrate of peat, vermiculite, and perlite at a ratio of 2:1:1. The pots were set up in a greenhouse with 16 h light and 8 h dark, and at temperatures of 24°C (day) and 18°C (night).

### 4.2 Dodder bridge construction

**Figure 9:**
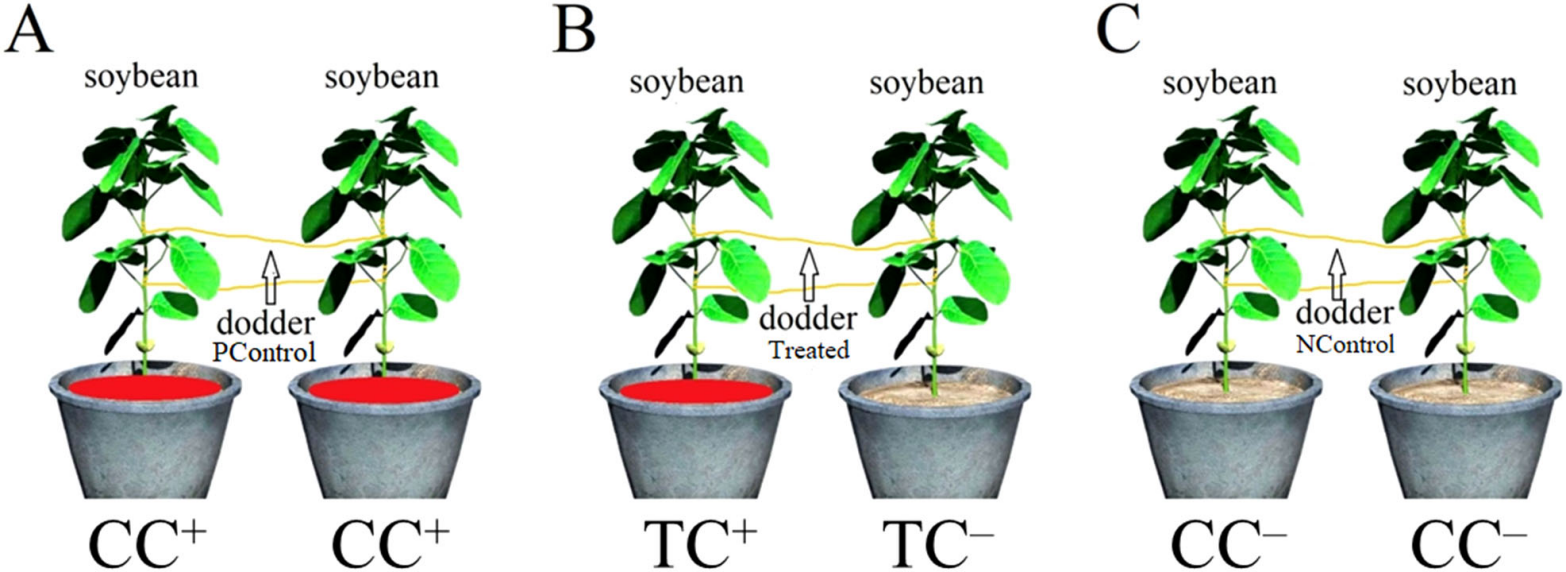
Experimental design. **(A)** Positive control. Both soybeans were treated with 200 mg/L CdSO_4_ (as CC^+^ soybean). Dodders were named as positive control dodders (PControl). **(B)** Experimental treatment. One soybean was treated with 200 mg/L CdSO_4_ (as TC^+^ soybean) and the other was treated with ddH_2_O (as TC^–^ soybean). Dodders were named as Treated dodders. **(C)** Negative control. Both soybeans were treated with ddH_2_O (as CC^–^ soybean). Dodders were named as negative control dodders (NControl).

Stems of *C. gronovii* were collected from the field of Taizhou University, Taizhou City, China. After 20 days of planting, when the donor soybean reached a length of 20 cm, approximately 10−15 cm long stems were used to parasitize the donor soybean. Seven days after parasitization, one recipient soybean was placed next to the donor soybean (at a distance of 10 cm), allowing the dodders to parasitize the neighboring soybean to form the dodder bridge. Donor soybeans were treated with 100mL 200 mg/L CdSO_4_ (as TC^+^ soybean), while the recipient soybean was left without CdSO_4_ treatment (as TC^–^ soybean). Both donor and recipient soybeans treated with CdSO_4_ (as CC^+^ soybean) and ddH_2_O (as CC^–^ soybean) were set up as positive and negative controls, respectively. Then, 100mL 200 mg/L CdSO_4_ was directly added into the soil. The experimental design is shown in **Figure 9**. Each group in this experiment has four replicates for each time point.

### 4.3 Sample collection and Cd^2+^ content measurement

After Cd^2+^ treatment for 2, 12, 24, and 48 h, samples of soil, soybean leaves, stem, root, and dodders were collected and dried at 70 °C for 48 h. Dried tissues were then ground into fine powder. Cd^2+^ content was determined using inductively coupled plasma-mass spectrometry (PerkinElmer, Massachusetts, America).

The third layer of soybean leaves from top to bottom was collected, washed with water, dried, and stored in liquid nitrogen for RNA-sequencing, each group has three replicates for each time point.

### 4.4 RNA-seq and data analysis

Total RNA was extracted using a total RNA Extraction Kit (Trizol, Sangon Biotechnology, Shanghai, China) following the manufacturer’s instructions. RNA integrity was assessed using the RNA Nano 6000 Assay Kit of the Bioanalyzer 2100 system (Agilent Technologies, CA, USA). Total RNA library was constructed and mRNA was enriched by oligo (dT) magnetic beads. The initial quantification of RNA was performed using Qubit 2.0 fluorometer. To ensure high-quality data for analysis, raw data were processed to remove reads containing joints, reads with ambiguous bases (N), and low-quality reads (defined as those with more than 50% of their length having Qphred scores ≤ 20). Based on the genome sequences of soybean to assemble the transcripts and identify DEGs. *C. gronovii* reads were analysis by *de novo* transcriptome assembly. Trinity (version 2.0.6) (Trinity Technologies, Irvine, CA, USA) was used for *de novo* assembly of the clean reads from the obtained samples. Gene function was annotated using the following databases: Nr (NCBI non-redundant protein sequence), Nt (NCBI non-redundant nucleotide sequence), Pfam (Protein family), KOG/COG (Clusters of Orthologous Genes), Swiss-Prot (a manually annotated and reviewed protein sequence database), KO (KEGG Ortholog database) and GO (Gene Ontology). The GO functional annotation was derived by comparing transcripts with the Swiss-Prot and TrEMBL databases, while Kyoto Encyclopedia of Genes and Genomes (KEGG) annotation information were obtained through KAAS acquisition. Differential expression analysis between two conditions or groups was conducted using DESeq2 R package (1.20.0). Genes with an adjusted p-value < 0.05 and a fold change ≥ 1 found by DESeq2 were identified as differentially expressed. To identify the mobile soybean mRNAs, the RNA-seq data from *C. gronovii* were mapped to soybean genome.

### 4.5 RT-qPCR validation

Six genes were selected for reverse transcription-quantitative polymerase chain reaction (RT-qPCR) validation. Primers were designed using primer5 software, and 60S Ribosomal Subunit gene served as the internal reference gene for the normalization of gene expression. The pre-extracted RNA was reverse transcribed into cDNA using a two-step RT-qPCR kit (Vazyme Biotechnology Co., Ltd., Nanjing, China) based on hiscript II reverse transcriptase. The amplification system was constructed using chamq universal SYBR qPCR Master Mix (Nanjing Visai Biotechnology Co., Ltd., China) and placed in CFX connect (Bio-rad Laboratories Inc., Hercules, CA, USA) for RT-qPCR. The relative expression of genes was measured using the 2^−ΔΔ^Ct method. The corrplot software package in R (3.6.1) was used for correlation analysis to verify the reliability of transcriptome data. Origin Pro software (version 8.0) was used to draw graphs based on data. Three technical replicates were used for each gene involved in the validation. Three biological replicates were used for each group at each time point.

### 4.6 Statistical data analysis

One-way ANOVA was conducted to test the effect of Cd^2+^ in different tissues by using IBM SPSS Statistics 20. Figures were drawn using Origin 2022.

## 5 Conclusion

**Figure 10:**
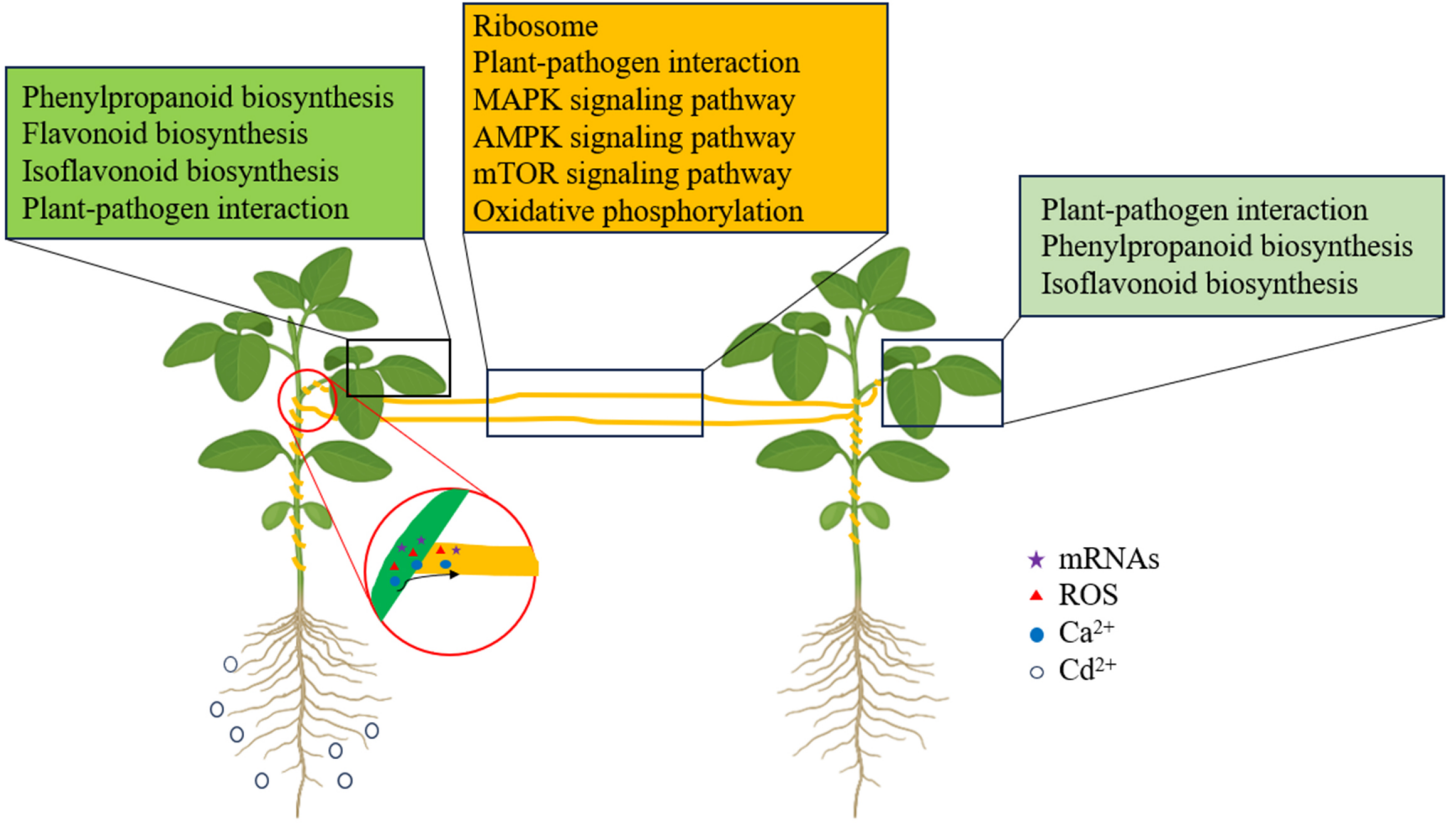
The schematic of the transcriptomic changes induced by Cd^2+^ addition in donor soybean, dodder bridge and neighboring recipient soybean. The key pathways enriched in donor soybean, dodder bridge and neighboring recipient soybean are listed in green, ochre and light green boxes. The red circles indicate the transfer of systemic signals from the haustoria between dodder and host soybean. Different symbols indicate different systemic signals. Small black circles in the left indicate Cd^2+^ addition.

Our study is the first to primarily investigate the direct changes in the transcriptome of donor soybean and indirect changes in the recipient soybean connected by dodder bridges induced by Cd^2+^ addition, and to predict several potential systemic signals. We clearly found that Cd^2+^ treatment can rapidly and directly induce significant changes of transcriptomic expression in soybean leaves, but take a while to induce such changes in the recipient soybean leaves connected by the dodder bridge. The direct or indirect changes were all related with ‘Plant-pathogen interaction’, ‘phenylpropanoid biosynthesis’ and ‘isflavonoid biosynthesis’. Cd^2+^ indirectly induced changes in the dodder bridge, which were related to ‘oxidation-reduction process’, ‘oxidoreductase activity’, and ‘regulation of transcription’ pathways. ‘Plant-pathogen interaction’, ‘MAPK signaling pathway’, ‘AMPK signaling pathway’, ‘mTOR signaling pathway’, which were related to Ca^2+^ up-regulated (**Figure 10**). Also, the transfer of mRNA exists from soybean to dodder and dodder to soybean. The transferred mRNA, Ca^2+^ and ROS might play roles in the systemic signals induced by Cd^2+^ addition. However, further studies are needed to use a range of genetically deficient plant lines as host plants to investigate the indirect changes in the plants by integrating phenomics, transcriptomics, proteomics and metabolomics methods and to explore the actual systemic signals induced and the processes that may be involved.

## Data Availability

The datasets presented in this study can be found in online repositories. The names of the repository/repositories and accession number(s) can be found in the article/supplementary material.

## Supplementary material

Supplementary Figure 1 The content of Cd in the root **(A)** and stem **(B)** of CC^+^, TC^+^, TC^–^, and CC^–^ soybeans at 2 h, 12 h, 24 h, and 48 h, respectively.

Supplementary Figure 2 The KEGG pathways enriched among the up-regulated DEGs between CC^+^ and CC^–^ at 2 h, 12 h, 24 h, and 48 h, respectively. Color of the point represents size of p-value. The number of differential genes included in each pathway is expressed by the point’s size.

Supplementary Figure 3 The KEGG pathways enriched among the up-regulated DEGs between TC^–^ and CC^–^ at 2 h, 12 h, 24 h, and 48 h, respectively. Color of the point represents size of p-value. The number of differential genes included in each pathway is expressed by the point’s size.

Supplementary Figure 4 The KEGG pathways enriched among the up-regulated DEGs between treated dodders and negative control dodders at 2 h, 12 h, 24 h, and 48 h, respectively. Color of the point represents size of p-value. The number of differential genes included in each pathway is expressed by the point’s size.

Supplementary Figure 5 Validation of the expression profiles of six genes by RT-qPCR. FPKM:Fragments Per Kilobase Per Million Reads. The left ordinate represents the FPKM, which is presented in the form of a line graph. The right ordinate represents the value obtained by fluorescence quantitative analysis of differentially expressed genes, which is presented in the form of a bar chart. R represents the correlation coefficient between the two data sets. The error bars represent the standard deviations.

Supplementary Figure 6 The FPKM of WRKY31, WRKY33, WRKY40, and WRKY42 in TC^+^ and TC^–^ at 2 h, 12 h, 24 h, and 48 h, respectively. FPKM: Per Kilobase Per Million Reads. Different small letters indicate significant difference of FPKM between TC^+^ and TC^–^.

Supplementary Figure 7 Co-expression analysis of the DEGS (TC^+^ vs TC^–^) involved in the phenylpropanoid biosynthesis pathway and 4 WRKY genes. Blue nodes indicate genes involved in phenylpropanoid biosynthesis pathway. Yellow nodes indicate 4 WRKY genes. Black lines indicate positive correlations.

## References

[B1] Andrews C. Xu Y. T. Kirberger M. Yang J. J. (2021). Structural aspects and prediction of calmodulin-binding proteins. Inter. J. Mol. Sci. 22, 308. doi: 10.3390/ijms22010308 PMC7795363 33396740

[B2] Bi G. Z. Hu M. Fu L. Zhang X. J. Zuo J. R. Li J. Y.. (2022). The cytosolic thiol peroxidase PRXIIB is an intracellular sensor for H2O2 that regulates plant immunity through a redox relay. Nat. Plant 8, 1160–1175. doi: 10.1038/s41477-022-01252-5 36241731

[B3] Birschwilks M. Haupt S. Hofius D. Neumann S. (2006). Transfer of phloem-mobile substances from the host plants to the holoparasite *Cuscuta* sp. J. Exp. Bot. 57, 911–921. doi: 10.1093/jxb/erj076 16467411

[B4] Chen B. J. W. Xu J. Wang X. Y. (2021). Trophic transfer without biomagnification of cadmium in a soybean-dodder parasitic system. Plants-basel. 10, 2690. doi: 10.3390/plants10122690 34961161 PMC8703755

[B5] Choi W. G. Miller G. Wallace I. Harper J. Mittler R. Gilroy S. (2017). Orchestrating rapid long-distance signaling in plants with Ca^2+^, ROS and electrical signals. Plant J. 90, 698–707. doi: 10.1111/tpj.13492 28112437 PMC5677518

[B6] Demidchik V. Maathuis F. Voitsekhovskaja O. (2018). Unravelling the plant signalling machinery: an update on the cellular and genetic basis of plant signal transduction. Funct. Plant Bio. 45, 1–8. doi: 10.1071/FP17085 32291017

[B7] Dong N. Q. Lin H. X. (2021). Contribution of phenylpropanoid metabolism to plant development and plant-environment interactions. J. Integrat. Plant Biol. 63, 180–209. doi: 10.1111/jipb.13054 33325112

[B8] Dorst H. J. M. V. Peters D. (1974). Some biological observations on pale fruit, a viroid-incited disease of cucumber. Eur. J. Plant Patho. 80, 85–96. doi: 10.1007/BF01980613

[B9] El-Esawi M. A. Elkelish A. Soliman M. Elansary H. O. Zaid A. Wani S. H. (2020). *Serratia marcescens* BM1 enhances cadmium stress tolerance and phytoremediation potential of soybean through modulation of osmolytes, leaf gas exchange, antioxidant machinery, and stress-responsive genes expression. Antioxidants. 9, 43. doi: 10.3390/antiox9010043 31947957 PMC7023057

[B10] Gong Z. H. Duan Y. Q. Liu D. M. Zong Y. Z. Zhang D. S. Shi X. R.. (2023). Physiological and transcriptome analysis of response of soybean (*Glycine max*) to cadmium stress under elevated CO_2_ concentration. J. Hazard. Mater. 448, 130950. doi: 10.1016/j.jhazmat.2023.130950 36860078

[B11] Haupt S. Oparka K. J. Sauer N. Neumann S. (2001). Macromolecular trafficking between *Nicotiana tabacum* and the holoparasite *Cuscuta reflexa* . J. Exp. Bot. 52, 173–177. doi: 10.1093/jexbot/52.354.173 11181727

[B12] Hettenhausen C. Lia J. Zhuang H. F. Sun H. H. Xu Y. X. Qi J. F.. (2017). Stem parasitic plant *Cuscuta australis* (dodder) transfers herbivory-induced signals among plants. PNAS. 114, e6703–e6709. doi: 10.1073/pnas.1704536114 28739895 PMC5559024

[B13] Hibberd J. M. Jeschke W. D. (2001). Solute flux into parasitic plants. J. Exp. Bot. 52, 2043–2049. doi: 10.1093/jexbot/52.363.2043 11559740

[B14] Hunter B. A. Johnson M. S. (1982). Food chain relationships of copper and cadmium in contaminated grassland ecosystems. Oikos. 38, 108–117. doi: 10.2307/3544572

[B15] Jiang L. J. Qu F. Li Z. H. Doohan D. (2013). Inter-species protein trafficking endows dodder (*Cuscuta pentagona*) with a host-specific herbicide-tolerant trait. New Phytol. 198, 1017–1022. doi: 10.1111/nph.12269 23550729

[B16] Kim G. LeBlanc M. L. Wafula E. K. Depamphilis C. W. Westwood J. H. (2014). Genomic-scale exchange of mRNA between a parasitic plant and its hosts. Science. 345, 808–811. doi: 10.1126/science.1253122 25124438

[B17] Köster P. DeFalco T. A. Zipfel C. (2022). Ca^2+^ signals in plant immunity. EMBO Journal. 41(12). doi: 10.15252/embj.2022110741 PMC9194748 35560235

[B18] Li S. L. Zhang J. X. Liu H. Liu N. Shen G. J. Zhuang H. F.. (2020). Dodder-transmitted mobile signals prime host plants for enhanced salt tolerance. J. Exp. Bot. 71, 1171–1184. doi: 10.1093/jxb/erz481 31665509 PMC6977188

[B19] Li Y. N. Tan M. T. Wu H. F. Zhang A. Y. Xu J. S. Meng Z. J.. (2023). Transfer of Cd along the food chain: The susceptibility of *Hyphantria cunea* larvae to *Beauveria bassiana* under Cd stress. J. Hazard. Mater. 453, 131420. doi: 10.1016/j.jhazmat.2023.131420 37084517

[B20] Luo F. Zhu D. Sun H. C. Zou R. Duan W. J. Liu J. X.. (2023). Wheat Selenium-binding protein TaSBP-A enhances cadmium tolerance by decreasing free Cd^2+^ and alleviating the oxidative damage and photosynthesis impairment. Front. Plant Sci. 14. doi: 10.3389/fpls.2023.1103241 PMC9941557 36824198

[B21] Marcone C. Ragozzino A. Seemuller E. (1997). Dodder transmission of alder yellows phytoplasma to the experimental host *Catharanthus roseus* (periwinkle). For. Pathol. 27, 347–350. doi: 10.1111/j.1439-0329.1997.tb01449.x

[B22] Mohanta T. K. Bashir T. Hashem A. Abd Allah E. F. Khan A. L. Al-Harrasi A. S. (2018). Early events in plant abiotic stress signaling: interplay between calcium, reactive oxygen species and phytohormones. J. Plant Growth Regul. 37, 1033–1049. doi: 10.1007/s00344-018-9833-8

[B23] Mower J. P. Stefanovic S. Hao W. Gummow J. S. Jain K. Ahmed D.. (2010). Horizontal acquisition of multiple mitochondrial genes from a parasitic plant followed by gene conversion with host mitochondrial genes. BMC Biol. 8, 150–166. doi: 10.1186/1741-7007-8-150 21176201 PMC3022774

[B24] Qiu Q. Wang Y. T. Yang Z. Y. Xin J. L. Yuan J. G. Wang J. B.. (2011). Responses of different Chinese flowering cabbage (*Brassica parachinensis* L.) cultivars to cadmium and lead exposure: screening for Cd+Pb pollution-safe cultivars. Clean-soil. 39, 925–932. doi: 10.1002/clen.201000275

[B25] Revill M. J. W. Stanley S. Hibberd J. M. (2005). Plastid genome structure and loss of photosynthetic ability in the parasitic genus *Cuscuta* . J. Exp. Bot. 56, 2477–2486. doi: 10.1093/jxb/eri240 16061507

[B26] Song J. Bian J. G. Xue N. Xu Y. X. Wu J. Q. (2022). Inter-species mRNA transfer among green peach aphids, dodder parasites, and cucumber host plants. Plant Divers. 44, 1–10. doi: 10.1016/j.pld.2021.03.004 35281124 PMC8897176

[B27] Song W. L. Zhang S. Y. Li Q. Xiang G. S. Zhao Y. Wei F.. (2024). Genome-wide profiling of WRKY genes involved in flavonoid biosynthesis in *Erigeron breviscapus* . Front. Plant Sci. 15. doi: 10.3389/fpls.2024.1412574 PMC11184973 38895611

[B28] Srivastava S. Tripathi R. D. Dwivedi U. N. (2004). Synthesis of phytochelatins and modulation of antioxidants in response to cadmium stress in *Cuscuta reflexa*-an angiospermic parasite. J. Plant Physiol. 161, 665–674. doi: 10.1078/0176-1617-01274 15266713

[B29] Sterckeman T. Thomine S. (2020). Mechanisms of cadmium accumulation in plants. Crit. Rev. Plant Sci. 39, 322–359. doi: 10.1080/07352689.2020.1792179

[B30] Tabassum N. Blilou I. (2022). Cell-to-cell communication during plant-pathogen interaction. Mol. Plant-Microbe Inter. 35, 98–108. doi: 10.1094/MPMI-09-21-0221-CR 34664986

[B31] Thornton I. Webb J. S. (1980). Trace elements in soils and plants. In: Blaxter K. (eds) Food Chain Nutr. Springer, Dordrecht. doi: 10.1007/978-94-011-7336-0_12

[B32] Vurro E. Ruotolo R. Ottonello S. Elviri L. Maffini M. Falasca G.. (2011). Phytochelatins govern zinc/copper homeostasis and cadmium detoxification in *Cuscuta campestris* parasitizing *Daucus carota* . Environ. Exp. Bot. 72, 26–33. doi: 10.1016/j.envexpbot.2010.04.017

[B33] Westwood H. J. Kim G. (2017). RNA mobility in parasitic plant-host interactions. RNA Biol. 14, 450–455. doi: 10.1080/15476286.2017.1291482 28277936 PMC5411121

[B34] Wu J. (2018). MiRNAs as a secret weapon in the battle field of haustoria, the interface between parasites and host plants. Mol. Plant 11, 354–356. doi: 10.1016/j.molp.2018.02.004 29462721

[B35] Wurzinger B. Mair A. Pfister B. Teige M. (2011). Cross-talk of calcium-dependent protein kinase and MAP kinase signaling. Plant Signal. Behav. 6, 8–12. doi: 10.4161/psb.6.1.14012 21248475 PMC3121996

[B36] Xian P. Q. Yang Y. Xiong C. W. Guo Z. B. Alam I. He Z. H.. (2023). Overexpression of *Gm*WRKY172 enhances cadmium tolerance in plants and reduces cadmium accumulation in soybean seeds. Front. Plant Sci. 14. doi: 10.3389/fpls.2023.1133892 PMC10033887 36968408

[B37] Yang L. Perrera V. Saplaoura E. Apelt F. Bahin M. Kramdi A.. (2019). m5C methylation guides systemic transport of messenger RNA over graft junctions in plants. Curr. Bio. 29, 2465. doi: 10.1016/j.cub.2019.06.042 31327714

[B38] Yang S. J. Han X. Li J. Luan F. Zhang S. L. Han D. Z.. (2024). Oceanobacillus picturae alleviates cadmium stress and promotes growth in soybean seedlings. J. Hazard. Mater. 472, 124568. doi: 10.1016/j.jhazmat.2024.134568 38749246

[B39] Zagorchev L. Stöggl W. Teofanova D. Li J. M. Kranner I. (2021). Plant parasites under pressure: effects of abiotic stress on the interactions between parasitic plants and their hosts. Int. J. Mol. Sci. 22, 7418. doi: 10.3390/ijms22147418 34299036 PMC8304456

[B40] Zhang J. X. Li S. L. Li W. X. Feng Z. R. Zhang S. H. Zheng X. J.. (2024). Large-scale interplant exchange of macromolecules between soybean and dodder under nutrient stresses. Plant Divers. 46, 116–125. doi: 10.1016/j.pld.2023.11.00 38343599 PMC10851303

[B41] Zhang J. X. Xu Y. X. Xie J. Zhuang H. F. Liu N. Shen G. J.. (2021). Parasite dodder enables transfer of bidirectional systemic nitrogen signals between host plants. Plant Physiol. 185, 1395–1410. doi: 10.1093/plphys/kiaa004 33793912 PMC8133666

