# Supplementary figures and images for: Transcriptomic changes in donor soybean, dodder bridge, and the connected recipient soybean induced by cadmium addition
Source: Front Plant Sci. 2025 Apr 17;16:1567412. doi: 10.3389/fpls.2025.1567412 (PMC12044426; doi:10.3389/fpls.2025.1567412)

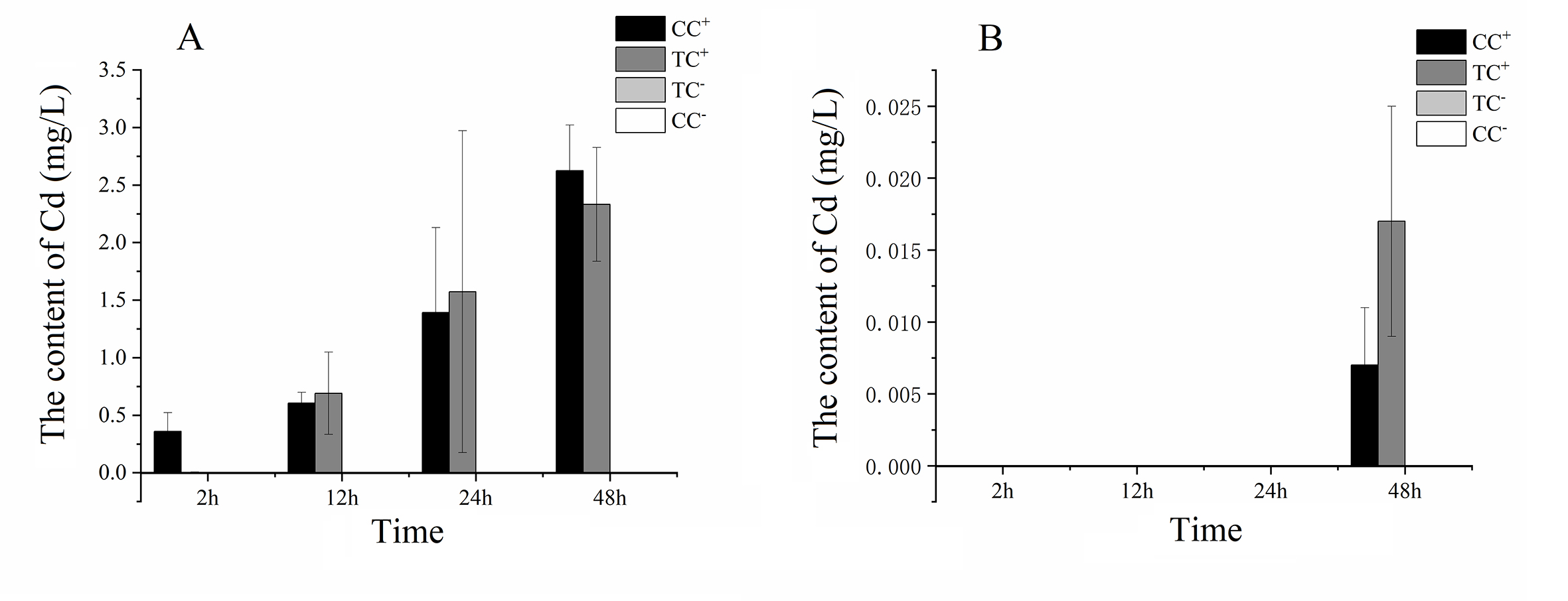

Supplement: Supplementary Figure 1 — The content of Cd in the root (A) and stem (B) of CC+, TC+, TC–, and CC– soybeans at 2 h, 12 h, 24 h, and 48 h, respectively. [file Image1.jpeg]

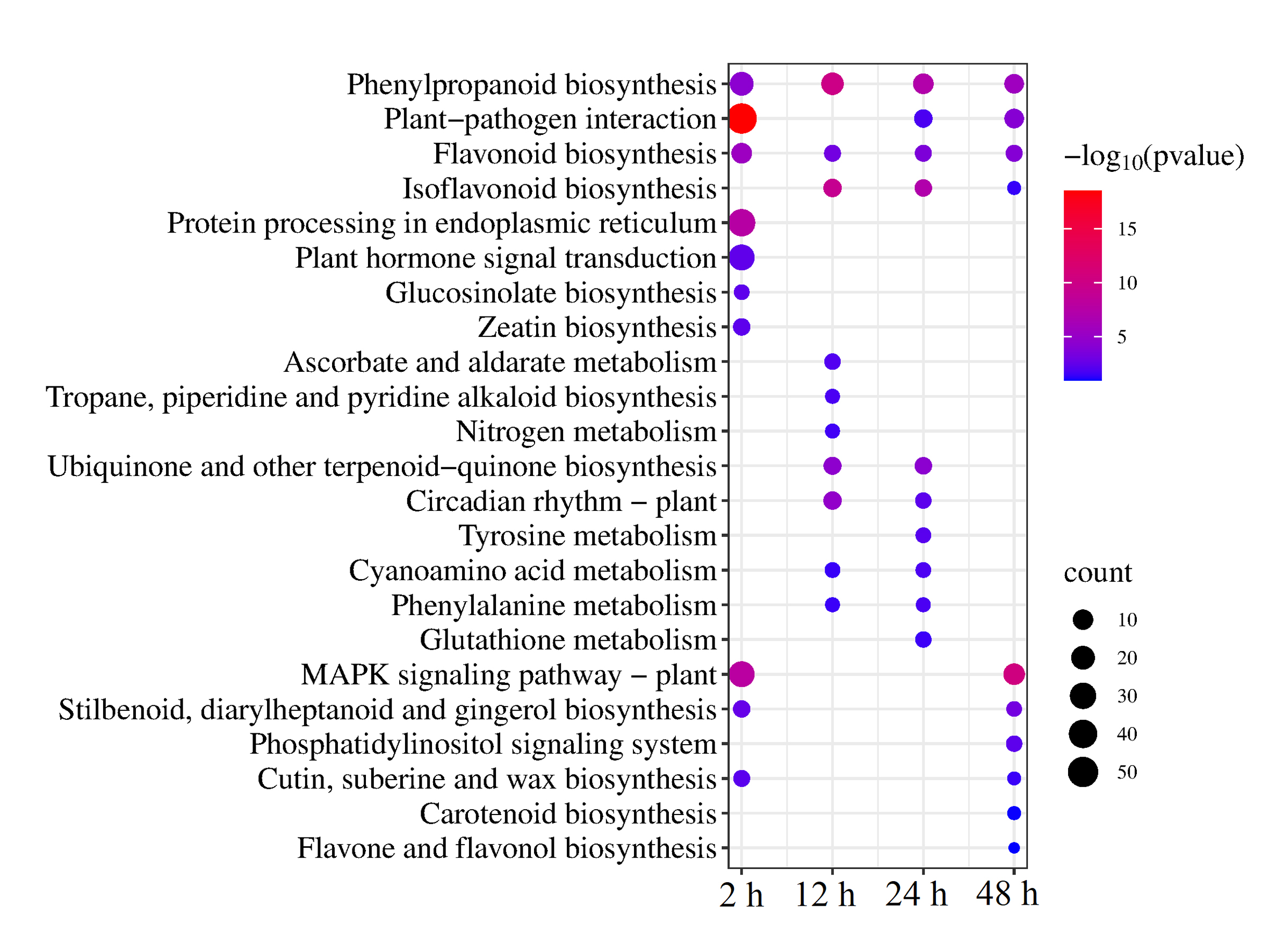

Supplement: Supplementary Figure 2 — The KEGG pathways enriched among the up-regulated DEGs between CC+ and CC– at 2 h, 12 h, 24 h, and 48 h, respectively. Color of the point represents size of p-value. The number of differential genes included in each pathway is expressed by the point’s size. [file Image2.jpeg]

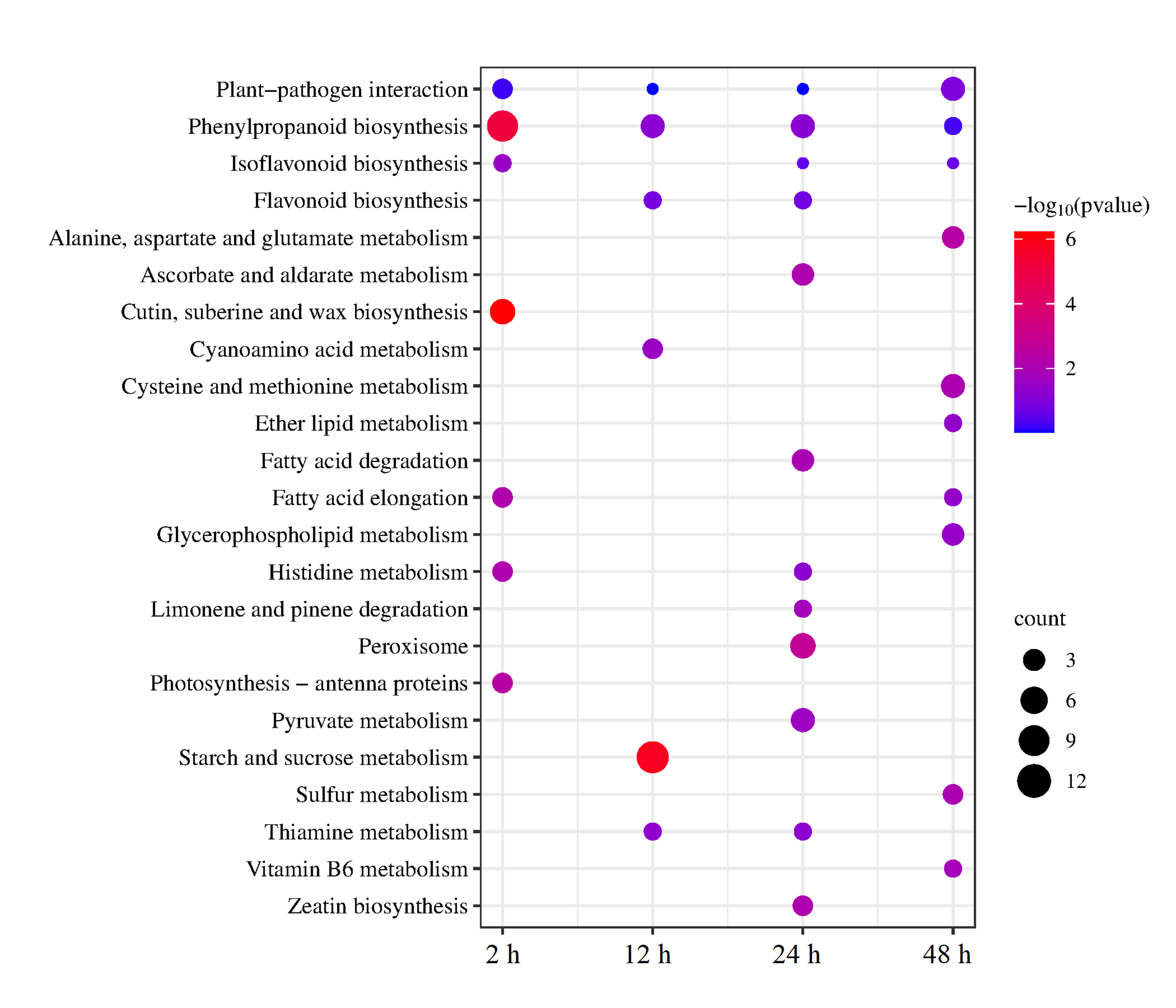

Supplement: Supplementary Figure 3 — The KEGG pathways enriched among the up-regulated DEGs between TC– and CC– at 2 h, 12 h, 24 h, and 48 h, respectively. Color of the point represents size of p-value. The number of differential genes included in each pathway is expressed by the point’s size. [file Image3.jpeg]

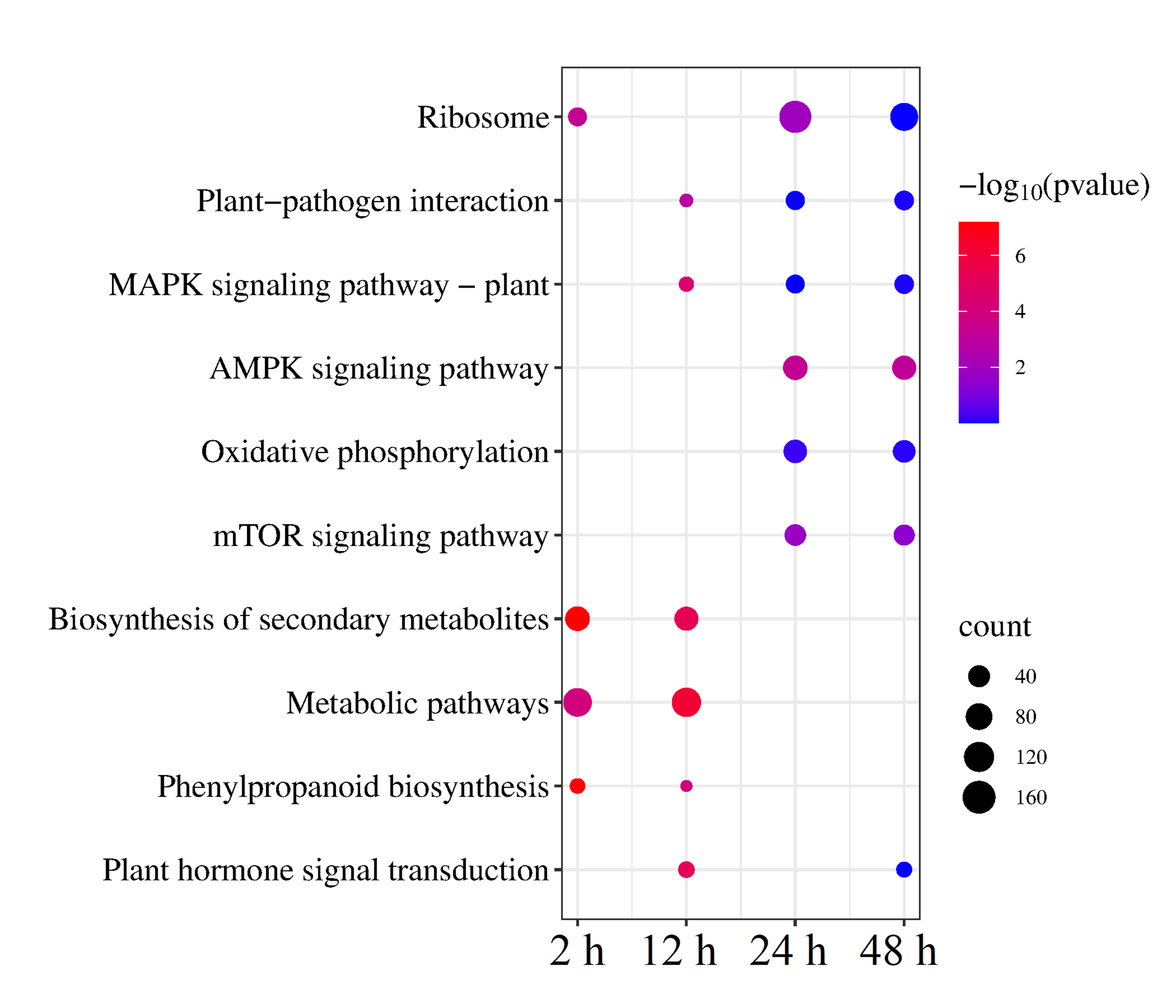

Supplement: Supplementary Figure 4 — The KEGG pathways enriched among the up-regulated DEGs between treated dodders and negative control dodders at 2 h, 12 h, 24 h, and 48 h, respectively. Color of the point represents size of p-value. The number of differential genes included in each pathway is expressed by the point’s size. [file Image4.jpeg]

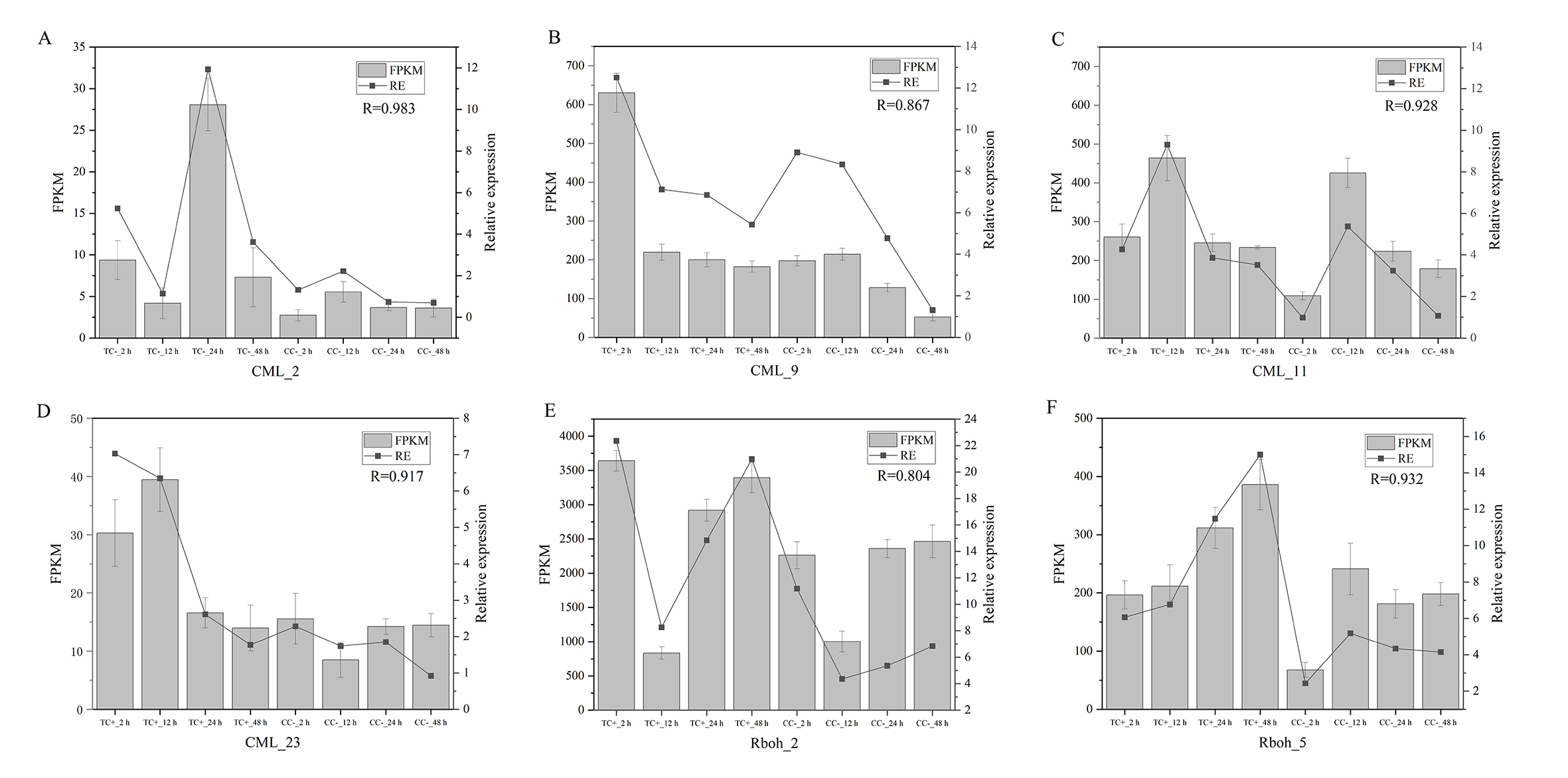

Supplement: Supplementary Figure 5 — Validation of the expression profiles of six genes by RT-qPCR. FPKM:Fragments Per Kilobase Per Million Reads. The left ordinate represents the FPKM, which is presented in the form of a line graph. The right ordinate represents the value obtained by fluorescence quantitative analysis of differentially expressed genes, which is presented in the form of a bar chart. R represents the correlation coefficient between the two data sets. The error bars represent the standard deviations. [file Image5.jpeg]

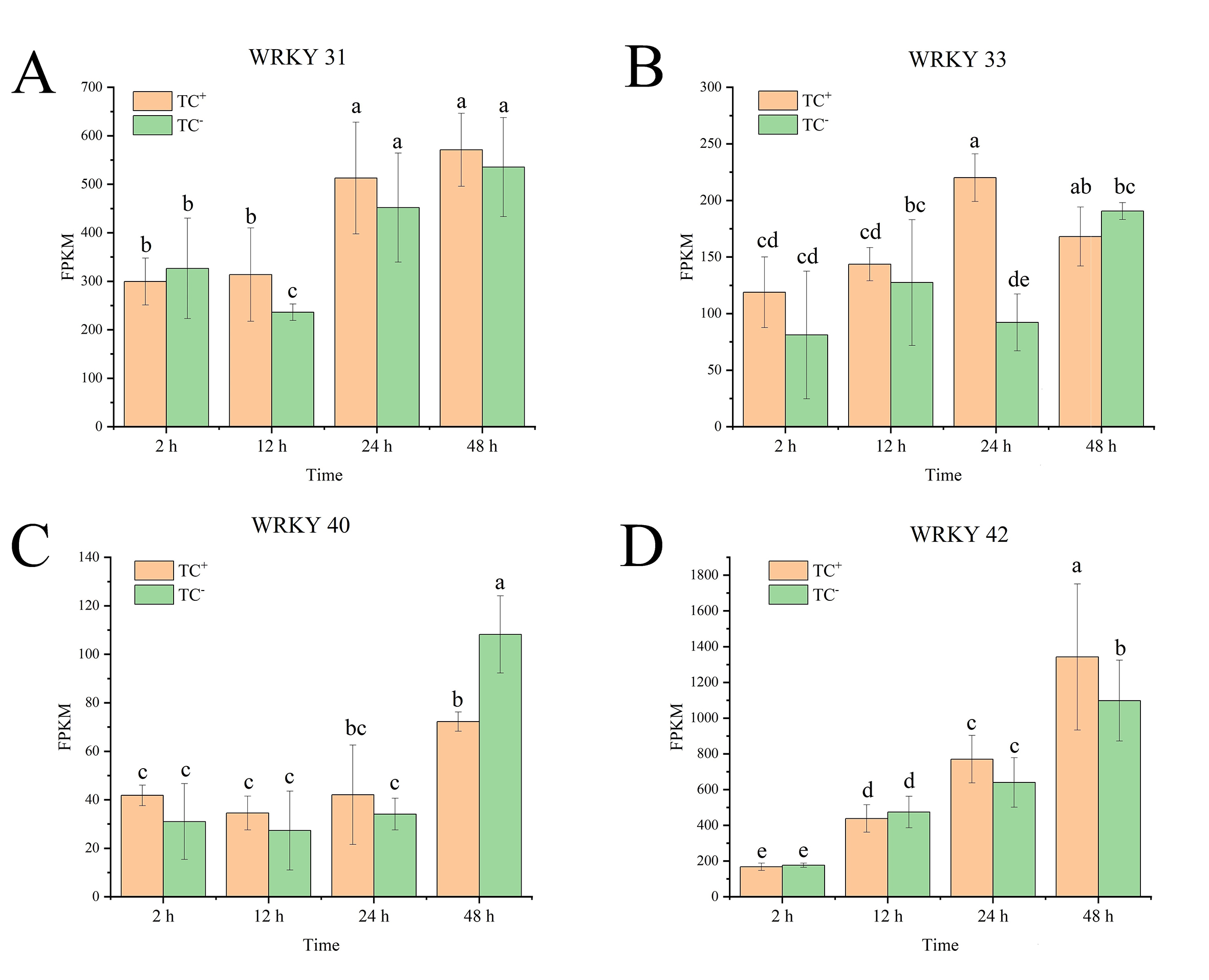

Supplement: Supplementary Figure 6 — The FPKM of WRKY31, WRKY33, WRKY40, and WRKY42 in TC+ and TC– at 2 h, 12 h, 24 h, and 48 h, respectively. FPKM: Per Kilobase Per Million Reads. Different small letters indicate significant difference of FPKM between TC+ and TC–. [file Image6.jpeg]

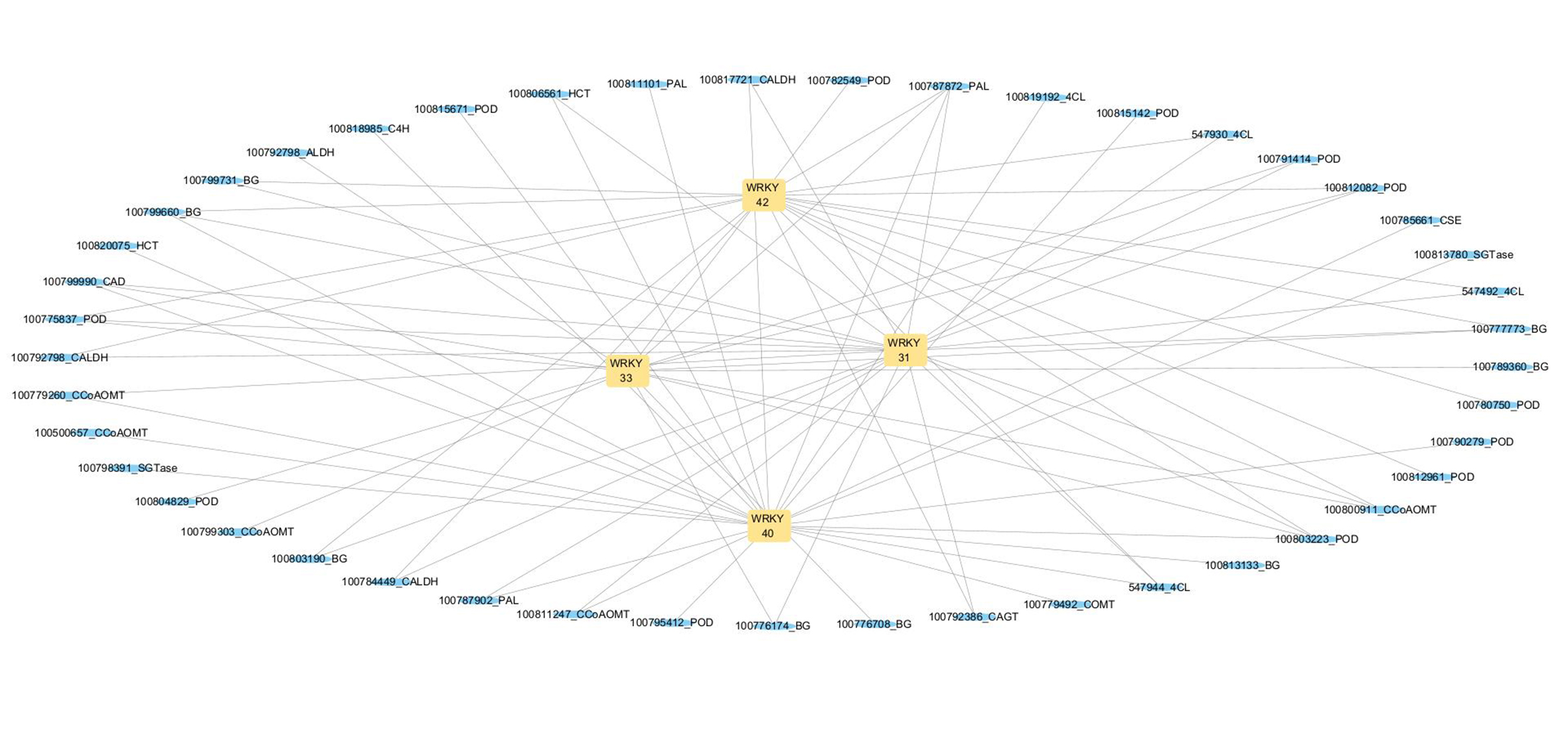

Supplement: Supplementary Figure 7 — Co-expression analysis of the DEGS (TC+ vs TC–) involved in the phenylpropanoid biosynthesis pathway and 4 WRKY genes. Blue nodes indicate genes involved in phenylpropanoid biosynthesis pathway. Yellow nodes indicate 4 WRKY genes. Black lines indicate positive correlations. [file Image7.jpeg]
